# Current Therapeutic Strategies for Patients with Hypopharyngeal Carcinoma: Oncologic and Functional Outcomes

**DOI:** 10.3390/jcm12031237

**Published:** 2023-02-03

**Authors:** Alexandre Bozec, Gilles Poissonnet, Olivier Dassonville, Dorian Culié

**Affiliations:** Institut Universitaire de la Face et du Cou, Centre Antoine Lacassagne, Université Côte d’Azur, 06103 Nice, France

**Keywords:** hypopharynx, squamous cell carcinoma, total laryngectomy, larynx preservation, survival, functional outcomes

## Abstract

Hypopharyngeal cancer is usually diagnosed at an advanced stage and is associated with a high risk of recurrence and poor survival rates. Although they differ greatly in terms of prognosis, hypopharyngeal cancers are usually treated together with laryngeal cancers in clinical trials. Therefore, there are very few studies that focus specifically on patients with hypopharyngeal carcinoma. As a result, the therapeutic management of these patients is highly debated, and their clinical outcomes are poorly reported. The aim of this review is therefore to discuss the current therapeutic options in patients with hypopharyngeal carcinoma and their oncologic and functional outcomes. Patients with early-stage tumors can be treated either by conservative surgery (including transoral robot-assisted surgery) or by RT alone. However, most patients are diagnosed with locally advanced tumors that cannot be treated surgically without total laryngectomy. In this situation, the critical issue is to select the patients eligible for a larynx preservation therapeutic program. However, radical surgery with total laryngectomy still plays an important role in the management of patients with hypopharyngeal carcinoma, either as the primary treatment modality (T4 resectable primary tumor, contraindication to larynx preservation therapies) or, more commonly, as salvage treatment.

## 1. Introduction

The Global Burden of Disease study estimated that 890,000 new head and neck cancers (HNCs) occurred worldwide in 2017, representing 5.3% of all cancers (excluding nonmelanoma skin cancers) [1,2]. Hypopharyngeal carcinoma (HC) accounts for approximately 15% of all HNCs and has a poor prognosis [2,3]. HC is a very aggressive malignancy characterized by submucosal tumor spread, perineural and vascular invasion, high risk of lymph node involvement, and metastasis [3,4,5,6,7,8]. HC usually occurs in patients with high tobacco and alcohol use, concomitant diseases, nutritional deficiencies, poor general health, and frequent psychosocial problems, making multidisciplinary management of these patients a real challenge [9,10,11,12]. Five-year overall survival (OS) rates close to 25% are commonly reported for patients with locally advanced disease, which is the most common initial disease presentation [3,12]. HC and its treatment affect critical functions such as breathing, the voice, and swallowing, and have a major impact on patients’ quality of life (QoL) [13]. HC treatment is therefore complex, usually multimodal, and requires multidisciplinary collaboration, which is best achieved in specialized head and neck cancer institutes [14]. 

The therapeutic decision-making process is difficult and includes tumor stage, previous treatments, the patient’s general health and concomitant diseases, psychosocial aspects, and the patient’s preferences. A significant proportion of patients are not eligible for standard treatments, and up to 10% of them are diagnosed with a second synchronous tumor in the upper aerodigestive tract, lung, or esophagus, which complicates the choice of treatment [4]. Although they have a completely different prognosis, HCs are often included with laryngeal cancers in larynx preservation (LP) studies [15]. There are thus very few studies specifically dedicated to HC patients. These studies are mostly retrospective case series with low levels of evidence [3]. Consequently, the therapeutic management of HC patients remains controversial and varies widely between centers.

The aim of this review is therefore to discuss the current therapeutic options for HC patients and their oncological and functional outcomes. This review article is not a systematic review but provides an overview of the medical literature on this important topic. 

## 2. General Considerations

Compared with other head and neck tumors, HC patients are characterized by a higher degree of tobacco and alcohol-related comorbidities and a higher risk of synchronous and metachronous second primary cancer arising from the upper aerodigestive tract, lung, and esophagus [3,4,5,6,7,8]. In a recent monocentric study, Thakur et al. reported a 2.7% prevalence of second synchronous esophageal carcinoma among all HNC patients, which was significantly increased in patients with supraglottic and pyriform sinus carcinoma [4]. In a nationwide, population-based study to evaluate the risk of metachronous esophageal carcinoma in HNC survivors, Tseng et al. showed that patients with oropharyngeal or HC had a significantly higher risk of developing metachronous esophageal cancers than patients with oral or laryngeal cancers (10-year incidence rates: 3.3% and 0.9%, respectively; hazard ratio: 2.15; 95% confidence interval: 1.57–2.96) [5]. In a retrospective Japanese study on 2011 HNC patients, Iwatsubo et al. showed that HC was independently associated with a higher risk of metachronous carcinoma even after multivariate analysis [6]. 

Of all HNC patients, HC patients also have the highest risk of distant metastases [7,8]. In a study that investigated the value of 18FDG PET/CT for detecting distant metastases in HNC patients, Haerle et al. showed that hypopharyngeal subsite and nodes in the inferior neck levels (IV/Vb) were independent predictors of distant metastases at diagnosis [7]. In a systematic review of the risk of developing distant metastases in HNC patients, Takes et al. showed that hypopharyngeal tumor location was among the most important predictive factors [8].

Due to the particular anatomical location of the tumor and the poor general health of patients, HNCs, similar to esophageal carcinomas, are associated with a significant risk of malnutrition at diagnosis, as well as during and after therapy [9,10,11,12]. In a recent study investigating the prognostic significance of the Geriatric Nutritional Risk Index (GNRI) in HNC patients, Nakayama et al. showed that patients with HC had the worse GNRI and that the GNRI was significantly associated with OS [10]. In a study on the risk of malnutrition in HNC patients, Yanni et al. found that hypopharyngeal tumor location was a significant predictor of malnutrition [11]. About one-third of HNC patients already suffer from malnutrition at the time of diagnosis, which increases during treatment, regardless of the therapeutic strategy [10,11,12]. Indeed, in a retrospective study of 53 HC patients enrolled in an induction chemotherapy (ICT)-based LP program, Bozec et al. reported a mean maximum weight loss during therapy of 8.7 ± 4.5 kg, with 17 (32%) patients requiring reactive enteral tube feeding [12]. This highlights the importance of early identification of malnourished patients and timely provision of appropriate nutritional support (nutritional counseling, oral nutritional supplements, enteral feeding via nasogastric or gastrostomy tubes, etc.). 

## 3. Management of Patients with Early-Stage Disease

Less than a quarter of HC patients are diagnosed with early-stage disease (i.e., T1-T2, N0 tumors) [3,13]. This can be explained, in contrast to glottic carcinomas, by the long asymptomatic or paucisymptomatic growth of these tumors and by their very strong propensity for lymph node invasion. In addition, the frequent psychosocial precariousness of HC patients may also explain delayed initial consultation. 

### 3.1. Primary Surgical Treatment

#### 3.1.1. Open Surgical Approaches

A small proportion of HC patients are eligible for primary conservative surgical treatment (i.e., without total laryngectomy) [16]. Various types of open surgical procedures have been used in selected patients with T1 or T2 HC [16,17,18,19]. Among these classic surgical procedures, lateral hypopharyngectomy (small tumors of the lateral pyriform sinus wall) and supraglottic hemipharyngolaryngectomy (small tumors of the aryepiglottic fold and medial pyriform sinus wall without involvement of the arytenoid) with ipsilateral neck dissection (ND) provide satisfactory and relatively consistent functional outcomes in selected patients [16,17,18,19]. In a retrospective analysis of 87 patients who underwent supraglottic hemipharyngolaryngectomy for T1 (16.1%) or T2 (83.9%) HC, Makeieff et al. reported persistent swallowing problems in only 6 patients [18]. The 5-year OS rate for patients with positive lymph nodes (N+) was 43.4% compared to 63.2% for patients with N-disease (*p* = 0.036) [18]. 

If the tumor extension is too close to the arytenoid cartilage to allow its preservation, surgical resection should include the entire aryepiglottic fold with the arytenoid cartilage. In this case, supracricoid hemipharyngolaryngectomy can be performed with satisfactory tumor control rates. However, recovery of adequate swallowing function is much less certain than in supraglottic resections, with frequent laryngeal penetrations/aspirations [16,17,18,19]. In an interesting study evaluating swallowing function in patients with vertical hemipharyngolaryngectomy for HC, Joo et al. showed that the rate of gastrostomy tube dependence was significantly higher after supracricoid hemipharyngolaryngectomy than after supraglottic hemipharyngolaryngectomy (35.7 vs. 0%, *p* = 0.014) [19]. Moreover, voice impairment is significant after this type of surgery.

#### 3.1.2. Transoral Surgical Approaches

Transoral surgery for HC includes transoral laser microsurgery (TLM) and transoral robotic surgery (TORS). These two technologies have been used to remove laryngeal and hypopharyngeal tumors, but recent developments seem to favor TLM for glottic lesions and TORS for supraglottic and hypopharyngeal lesions [20]. Compared with open surgical approaches, the same types of tumor resections can be performed but without removing a fragment of the thyroid cartilage and hyoid bone and without transecting the infrahyoid muscles. Consequently, TORS is associated with lower postoperative morbidity compared with open surgical procedures, including a lower rate of tracheostomies, faster recovery of swallowing function, and shorter length of stay [20,21]. In a multicenter study evaluating the oncologic and functional outcomes of TORS in 57 patients with pyriform sinus carcinoma, the French collaborative study group GETTEC reported a 12% rate of preventive tracheostomies (all successfully decannulated), oral feeding recovery in 93% of patients after a median of 5 days, and a median hospital stay of 10 days [21]. Two-year and five-year OS rates were 84% and 66%, respectively, with an organ preservation rate of 96% at the end of follow-up [21]. Similar to other TORS procedures, attention should be paid to the risk of postoperative bleeding [22]. Ipsilateral selective ND (levels II to IV) should be performed concurrently with primary tumor resection or as a separate procedure.

### 3.2. Nonsurgical Treatment 

In addition to surgical treatment, RT alone is another validated therapeutic option for patients with T1-2, N0 HC [23,24,25]. In a multi-institutional analysis of 115 patients with early-stage HC treated with definitive RT, Nakamura et al. reported 5-year OS and disease-specific survival (DSS) rates of 66.0% and 77.4%, respectively, for 95 patients without synchronous malignancies [23]. However, 65 patients (56.5%) had synchronous (*n* = 20) or metachronous (*n* = 45) cancers, and 10 of them died of a second primary cancer during the study period [23]. This highlights the particular risk of second primary malignancy in HC patients, which must be considered when making treatment decisions. In a retrospective monocentric study of 103 patients with T1-2 HC, Nakajima et al. showed 3-year OS and DSS rates of 70% and 79%, respectively, with 3-year local control rates of 87% for T1 and 83% for T2 disease [24]. Sixty patients developed synchronous or metachronous second primary carcinomas [24]. In another monocentric retrospective analysis of 123 patients with pyriform sinus carcinoma, Rabbani et al. found 5-year local control, DSS, and OS rates of 85%, 61%, and 35%, respectively [25]. The overall local control rate with a functional larynx was 83% [25]. Taken together, these results suggest that satisfactory local control and survival rates for early-stage HC can be achieved with definitive RT.

### 3.3. Choice of the Therapeutic Strategy 

**Figure 1:**
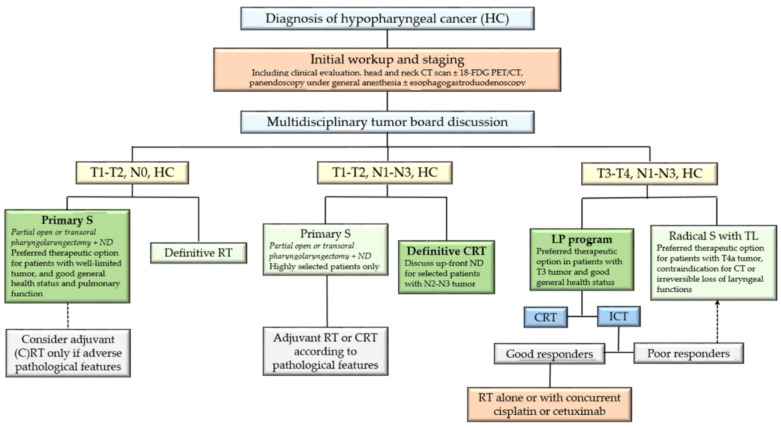
Therapeutic management of patients with nonmetastatic resectable hypopharyngeal carcinoma (HC). CT: computed tomography; PET: positron emission tomography; S: surgery; ND: neck dissection; RT: radiation therapy; CRT: chemoradiation therapy; ICT: induction chemotherapy; TL: total laryngectomy.

Comparable tumor control and survival rates are achieved in patients with early-stage HC by either surgery or definitive RT. Satisfactory functional outcomes are generally achieved, including restoration of swallowing function (complete oral nutrition without reliance on a feeding tube) [14,16]. In this context, the choice of the appropriate therapeutic strategy is complex and depends, in particular, on the tumor characteristics and extension (well or poorly demarcated lesions, ulcerative, infiltrating or exophytic tumors), the patient’s age, concomitant diseases (especially pulmonary function), and preferences [14]. Patients with good general health and adequate respiratory function who have an ulcerative, well-demarcated tumor suitable for transoral resection should preferably undergo primary transoral surgery with ipsilateral ND [14,16]. On the other hand, patients with a poorly demarcated tumor or severe comorbidities should be treated with definitive RT [16,23,24]. The high risk of metachronous development of a second primary tumor is a strong argument for reserving RT for future treatment and therefore favoring a primary surgical strategy in HC patients who are candidates for primary tumor resection (Figure 1) [3,4]. 

## 4. Management of Patients with T1-2, N1-3, M0 Tumors

Because of lymph node metastases, patients with T1-2, N1-3 HC should receive multimodal treatment [14]. Thus, patients referred to primary surgical treatment undergo postoperative RT or CRT, while patients referred to nonsurgical treatment receive 3-weekly cisplatin (100 mg/m^2^) concurrently with definitive RT [14]. In this context, open or transoral resection of the primary tumor should be considered only in well-selected cases when optimal postoperative swallowing function can be expected with certainty. Indeed, postoperative adjuvant RT or CRT will inevitably worsen patients’ functional outcomes, while the survival benefits of primary surgery are not well established [16]. Most T1-2, N1-3 HC patients are therefore referred to primary cisplatin-based CRT, while surgery is reserved as a salvage therapy.

Some studies have nevertheless investigated the role of upfront ND for patients with N2-3 HC [26,27,28]. These studies were retrospective analyses of case series and showed contrasting results [26,27,28]. Therefore, due to the lack of well-designed randomized trials, it is difficult to draw conclusions about the role of upfront ND in HC patients before definitive CRT [28].

Taken together, these data suggest that most patients with T1-2, N1-3 HC should be referred to definitive CRT (Figure 1). Primary surgical treatment should be reserved for well-selected patients when experienced surgeons can expect minimal postoperative morbidity. Upfront ND may be an interesting option for patients with N2-3 disease, but its specific role remains to be defined.

## 5. Management of Patients with T3-4, N1-3, M0 Tumors

### 5.1. Radical Surgery

#### 5.1.1. Oncologic Surgery

The vast majority of patients with T3-4 HC cannot be operated on without TL. Indeed, TL with partial or circular pharyngectomy and bilateral neck dissection is the standard surgical treatment for patients with locally advanced (T ≥ 3) HC [29]. However, with advances in surgical skills, open organ-preserving laryngopharyngeal surgical techniques have been developed for highly selected patients [30,31,32]. In a recent study evaluating the oncologic and functional outcomes of supracricoid hemipharyngectomy in 23 patients with T3-4 HC, Xu et al. reported that oral realimentation and decannulation were achieved within 6 months in 82.6% and 87.0% of patients, respectively [31]. Five-year OS and disease-free survival (DFS) rates were 63.8% and 60.3%, respectively [31]. When tumor resection is extended to the hemicricoid cartilage, some authors have investigated complex pharyngolaryngeal reconstruction with a sensory free radial forearm flap and a free cartilage graft [32]. In very selected and motivated patients, functional reconstruction can be achieved after this type of surgery. Swallowing function recovery without laryngeal penetration and aspiration is, however, uncertain and requires a long and demanding rehabilitation period [32]. This type of surgery cannot therefore be considered as a standard surgical procedure for patients with T3-4 HC.

In radical tumor resection with TL in T3-4 HC patients, the main question for the surgeon is the extent of pharynx to be resected, from partial to circular pharyngectomy, and consequently the choice of surgical technique required for pharyngeal closure or reconstruction [33,34]. This question must be clarified before surgery with a precise description of the tumor extension by endoscopic and imaging procedures. Attention should be paid to involvement of the posterior pharyngeal wall, retrocricoid area, and pharyngoesophageal junction. Gross tumor involvement of these critical anatomic structures requires circular (or near-circular) resection of the pharynx, whereas tumor extension to the cervical esophagus may also require total esophagectomy [34]. Resection of the pharynx may also be extended to the oropharynx if the tumor extends to the lateral/posterior oropharyngeal wall or the tongue base, which may affect postoperative swallowing function.

In patients with previously untreated locally advanced HC, bilateral ND and primary tumor ablation should be performed simultaneously. Because most HC patients have multiple and/or large neck metastases, radically modified or extended ND (resection of cranial nerves X or XII or external carotid artery, etc.) is often required. When discussing the optimal therapeutic strategy, it should be considered that N stage has a significant impact on the overall prognosis of HC patients [35,36]. In a recent retrospective study in patients with locally advanced HC, Mattei et al. showed, after multivariate analysis, that N stage ≥ 2 was significantly associated with worse DSS (*p* = 0.01) and swallowing outcomes (Dysphagia Outcome and Severity Scale (DOSS); *p* = 0.02) [35]. In another recent retrospective study in patients with advanced laryngeal and HC after primary TL, Grasl et al. found that lymph node ratio was an independent predictor of OS (*p* = 0.004), DSS (*p* = 0.009), and DFS (*p* = 0.044) in a multivariate analysis [36].

Oncologic and functional outcomes of radical surgery (i.e., with TL) vary widely depending on the primary tumor site. Even after primary radical surgery, patients with HC show worse survival and functional outcomes (swallowing and speech) than patients with laryngeal cancer [37]. In a retrospective study of 63 T4 laryngeal or HC patients who underwent radical surgery with TL, Roux et al. showed that hypopharyngeal tumor location had a negative independent effect on DSS (*p* = 0.005; OR = 10.8; CI 95%: 1.9–58.6) [37]. While 5-year OS rates above 50% are frequently reported in patients with locally advanced laryngeal cancer undergoing primary TL, 5-year OS rates close to 30% are generally found for patients with HC [3,35,37]. In a retrospective study analyzing treatment strategies in 217 patients with stage T3-4 laryngeal cancer, Mattei et al. found a 5-year OS rate of 28% in the 40 patients treated with primary radical surgery [35]. More encouraging results were reported by Chung et al. in a retrospective analysis of 266 patients with stage III /IV HC, 139 of whom underwent primary surgery [38]. The authors reported a 5-year OS rate of 45.3% in the surgical group. However, 42.1% of the patients included in this series had a T1-2 primary tumor, which was classified as locally advanced cancer based on N stage [38].

#### 5.1.2. Reconstructive Surgery

After TL with partial pharyngectomy, direct closure of the pharynx is performed, when possible, without stenosis. This is generally possible for lesions that do not involve the retrocricoid region or posterior pharyngeal wall [29]. A myofascial pectoralis major flap is applied to cover the pharyngeal sutures and reduce the risk of fistula formation in previously irradiated patients (salvage surgery) [39].

When direct closure is not possible, a soft tissue flap with a skin island sutured as a patch to the remaining pharyngeal mucosa can be a simple and reliable reconstruction method [39]. In this situation, pectoralis major or latissimus dorsi (preferred option in women) myocutaneous flaps are the most common techniques for partial pharyngeal reconstruction [29,39]. The skin island is sutured to the rest of the posterior pharyngeal wall, and the large muscular portion of the flap is used to cover the pharyngeal sutures and protect the neck vessels. Other pedicled soft tissue flaps such as the supraclavicular flap can be used for this indication [40].

The problem of hypopharyngeal reconstruction in the context of TL with circular pharyngectomy resulting in a circumferential pharyngeal defect is much more difficult [34]. In this situation, fasciocutaneous free flaps (radial forearm or anterolateral flaps) and jejunal free flaps are two standard reconstructive techniques [34,41,42]. The jejunal free flap has a lower risk of postoperative salivary fistula and pharyngoesophageal stenosis than the fasciocutaneous free flap, but exposes patients to the morbidity of laparotomy and requires a visceral surgeon to harvest the flap [42,43]. Unlike jejunal flaps, fasciocutaneous free flaps must be tubulated. Some authors use salivary bypass tubes to reduce the risk and severity of fistula formation [44]. While improved swallowing function has been reported with jejunal flaps compared with fasciocutaneous flaps, some authors have noted poorer outcomes in speech rehabilitation [42,45]. Although the natural secretion of the jejunal mucosa is an advantage for swallowing, it may affect the intelligibility of tracheoesophageal speech. Complete tubulization of pedicled myocutaneous flaps often results in stenosis at the pharyngoesophageal junction. The use of a U-shaped pectoralis major myocutaneous flap sutured directly to the prevertebral fascia has been reported but places the patient at high risk for salivary fistulas and stenosis [46].

In cases of large tumoral invasion of the cervical esophagus, complete esophagectomy should be performed in association with TL and circular pharyngectomy [28,47]. In this case, reconstruction is achieved by tubular gastroplasty (gastric pull-up) or colon interposition (coloplasty) [47]. Postoperative local and general complication and mortality rates are higher than after TL and circular pharyngectomy without esophagectomy, but long-term swallowing outcomes are satisfactory, with most patients achieving a full oral diet [47]. The gastric pull-up technique is the first reconstructive option in most centers but imposes dietary modifications (6–8 small meals/day). Successful voice restoration after secondary tracheoesophageal prosthesis (TEP) insertion has been reported in selected patients [48]. Indeed, in a recent retrospective study on seven patients who underwent TL with circular pharyngectomy and esophagectomy associated with gastric pull-up or colon interposition, Molteni et al. showed that an intelligible voice was restored in all patients with satisfactory patient-perceived voice-related and dysphagia-related QoL outcomes [48].

#### 5.1.3. Rehabilitation Measures

Voice restoration through TEP placement is recognized as the gold-standard voice rehabilitation technique in laryngectomized patients [49,50,51,52]. Neurocognitive deficiencies, depressive symptoms, lack of motivation, poor respiratory function, and pharyngoesophageal stenosis have been identified as significant predictors of voice rehabilitation failure and should be considered in the decision of TEP placement [49,50,51]. Indeed, in a recent study conducted in 48 laryngectomized patients after successful voice restoration using TEP, we showed that an anxiodepressive disorder (HADS global score ≥ 15) was reported by 15 (31%) patients and represented the main predictor of QoL and voice outcomes (VHI-10 score) [49]. In a retrospective study on 168 laryngectomized patients who underwent secondary TEP placement, Lavertu et al. found that, after multivariate analysis, only the presence of pharyngeal stricture was associated with a poorer-quality voice (*p* = 0.001) [50]. This may explain why patients undergoing circular pharyngeal resection, which is associated with a higher risk of pharyngeal stenosis, generally achieved worse voice outcomes than those undergoing TL with partial pharyngectomy.

Primary or secondary TEP insertion achieved similar long-term functional results, but primary TEP placement can naturally provide faster functional recovery [51,52]. However, due to an increased risk of postoperative fistula, secondary placement should be favored in cases of circular pharyngectomy with or without esophagectomy and when the pharyngeal suture inferior level is situated less than 2 cm from the TEP insertion site [51]. In this regard, we have previously shown in a series of 103 patients undergoing TL that hypopharyngeal tumors (*p* = 0.005), circular pharyngectomy (*p* = 0.003), and use of a pectoralis major myocutaneous flap for pharyngeal closure (*p* = 0.0003) were significantly associated with secondary TEP placement [51]. Similarly, in a retrospective analysis of 145 patients who underwent TL with primary or secondary TEP placement, Gitomer et al. concluded that extent of pharyngeal reconstruction, rather than radiation, may be more important in selection of TEP timing [52]. Speech rehabilitation should be initiated as soon as complete healing is obtained.

### 5.2. Larynx Preservation (LP) Approaches

In the 1990s, considerable research efforts were made to achieve effective LP in patients with locally advanced laryngeal or HC, using various combinations of RT and CT [15,53,54,55]. Several randomized prospective studies showed that, in well-selected patients, these innovative therapeutic regimens could achieve survival rates similar to primary radical surgery followed by adjuvant RT [15]. Two types of LP protocols were developed. The first one, which is generally favored in France and other Latin European countries, consisted of an induction CT (ICT) followed by RT in good responders to ICT. The second one, which is favored in North America and the United Kingdom, consisted of a definitive concurrent CRT [15].

The development of ICT with cisplatin plus 5-fluorouracil (PF) and the correlation between CT and RT sensitivity in previously untreated patients opened a new era of LP protocols for patients with locally advanced laryngeal or HC. The fundamental concept was to employ induction PF in order to select patients for subsequent treatment with either TL or RT according to tumor response to PF [15]. The first two trials (VALGSG for laryngeal cancer and EORTC 24891 for HC) concluded that such an approach could preserve nearly 60% of the larynx without deleterious impact on survival [53,54]. The EORTC 24,954 trial compared a sequential arm (SA = induction PF followed by a 70Gy-RT for the responders or a TL and postoperative RT for the nonresponders) and an alternating arm (AA = PF alternated with three 2-week courses of 20 Gy-RT for a total dose of 60 Gy) [55]. After a median follow-up of 10.2 years, 10-year survival with functional larynx (primary endpoint) and OS were similar in both arms (18.7% and 33.6% in SA versus 18.3% and 31.6% in AA) [55].

The GORTEC 2000-01 trial compared induction PF to induction PF plus docetaxel (TPF), both followed by RT in good responders in laryngeal and HC [56]. Two hundred and thirteen patients were treated with a median follow-up of 105 months. The 10-year LP rates were 70.3% (95% CI = 0.58 to 0.8) vs. 46.5% (95% CI = 0.31 to 0.63, *p* = 0.01) in the TPF and PF arms, respectively. The 10-year larynx dysfunction-free survival rates were 63.7% (95% CI = 0.52 to 0.74) vs. 37.2% (95% CI = 0.24 to 0.52, *p* = 0.001). However, OS and DFS were not statistically improved in the TPF vs. the PF arm [56]. These data along with those of other randomized studies comparing TPF with PF-based ICT in patients with locally advanced and/or unresectable HNC have confirmed the TPF regimen as the gold-standard ICT approach in HNC patients [15,56].

The RTOG 91-11 trial is an important study among all LP studies, but it only enrolled patients with laryngeal cancer [57]. Patients with stage III or IV glottic or supraglottic squamous cell carcinoma were randomly assigned to ICT (PF) followed by RT (control arm), concomitant CRT (cisplatin), or RT alone. There was no significant difference in 5-year laryngectomy-free survival between the patients treated with ICT (44%) and those treated with CRT (47%), both being superior to RT alone (34%). OS did not differ significantly, although there was a possibility of a worse outcome with concomitant relative to ICT (HR, 1.25; 95% CI, 0.98 to 1.61; *p* = 0.08). Concomitant CRT significantly improved the LP rate over ICT followed by RT (HR, 0.58; 95% CI, 0.37 to 0.89; *p* = 0.005) and over RT alone (*p* < 0.001). No difference in late effects was detected, but deaths not attributed to larynx cancer or treatment were higher with concomitant chemotherapy (30.8% vs. 20.8% with ICT and 16.9% with RT alone) [57].

Two phase II trials explored the role of cetuximab (E) for LP in laryngeal and HC [15]. The TREMPLIN trial compared RT+cisplatin (arm A) vs. RT+E (arm B) after TPF-based ICT [58]. There was no significant difference in LP at 3 months between arms A and B (95% and 93%, respectively), in larynx functional preservation (87% and 82%, respectively), and OS at 18 months (92% and 89%, respectively) [58]. The authors concluded that there was no evidence that one treatment was superior to the other or could improve the outcome reported with ICT followed by RT alone (GORTEC 2000-01 trial) [58]. The DeLOS-II trial tested the hypothesis that E added to ICT and RT improved laryngectomy-free survival [59]. Due to four therapy-related deaths among the first 64 randomized patients, 5-FU was omitted from ICT in the subsequent 112 patients. Despite being accompanied by an elevated frequency of adverse events, the ICT with TPF/TP plus E was feasible but showed no superiority to ICT with TPF/TP regarding laryngectomy-free survival and OS at 24 months [59].

**Figure 2:**
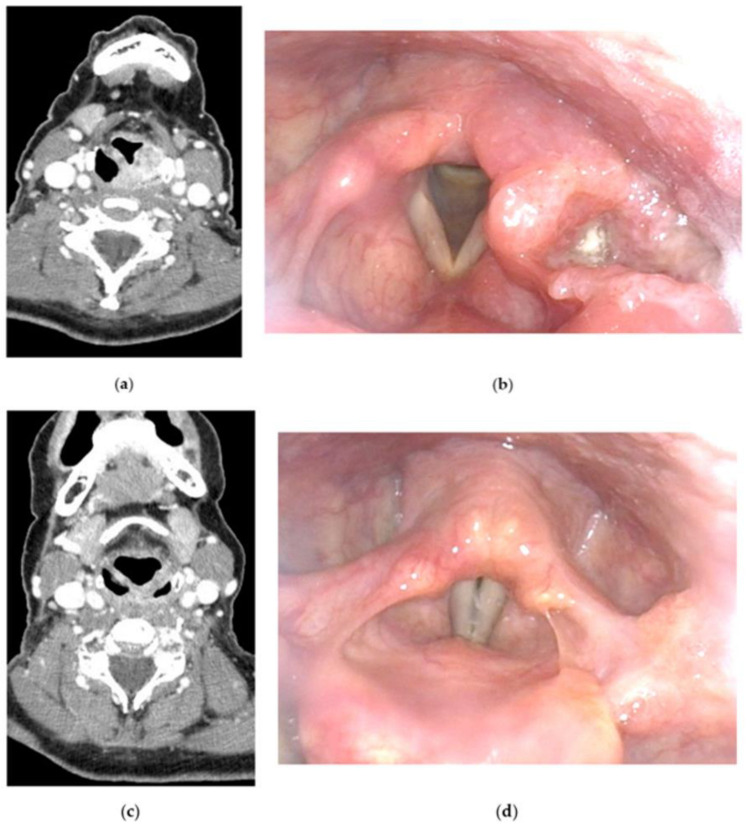
Induction chemotherapy (ICT)-based larynx preservation (LP) program for a 62-year-old female patient with a T3N0 hypopharyngeal carcinoma: (**a**) initial CT scan of the tumor; (**b**) initial nasofibroscopy showing a T3 tumor invading the left pyriform sinus, and the aryepiglottic fold; (**c**) post-ICT CT scan showing a near-complete response to ICT (2 cycles of TPF: docetaxel, cisplatin and 5-fluorouracil); (**d**) post-ICT nasofibroscopy showing a near-complete response to ICT with larynx mobility recovery.

To date, two approaches for LP have been validated: induction TPF followed by RT for laryngeal and HC and concurrent CRT for laryngeal carcinoma [15]. However, in clinical practice, concurrent cisplatin-based CRT is also used in HC patients as for patients with other locally advanced/unresectable HNC. An ongoing trial (SALTORL: ClinicalTrials.gov Identifier: NCT03340896) is comparing these two therapeutic approaches, induction TPF followed by RT in responders to ICT and definitive concurrent CRT, in patients with laryngeal or HC. The primary endpoint is 2-year larynx dysfunction-free survival [15]. For centers favoring an ICT-based LP strategy, the criteria chosen to define responders to ICT represent a critical issue [15,29]. Although a complete or quasi-complete response was required to pursue conservative treatment in the first LP studies, an objective partial response (response > 50%) was considered sufficient when associated with larynx remobilization (restoration of vocal cord mobility) in recent LP clinical trials (Figure 2) [15,29].

### 5.3. Choice of the Therapeutic Strategy

In patients with locally advanced HC, similarly to those with laryngeal cancer, an LP approach is favored when preservation of laryngeal functions (airway, voice, and swallowing) can be reasonably expected without compromising patient survival [15,29] (Figure 1). For this purpose, three main criteria should be met: (i) no major predictive factor of treatment failure; (ii) no contraindication for TPF-ICT or for concurrent cisplatin-based CRT; (iii) no irreversible loss of laryngeal function [15,29].

**Figure 3:**
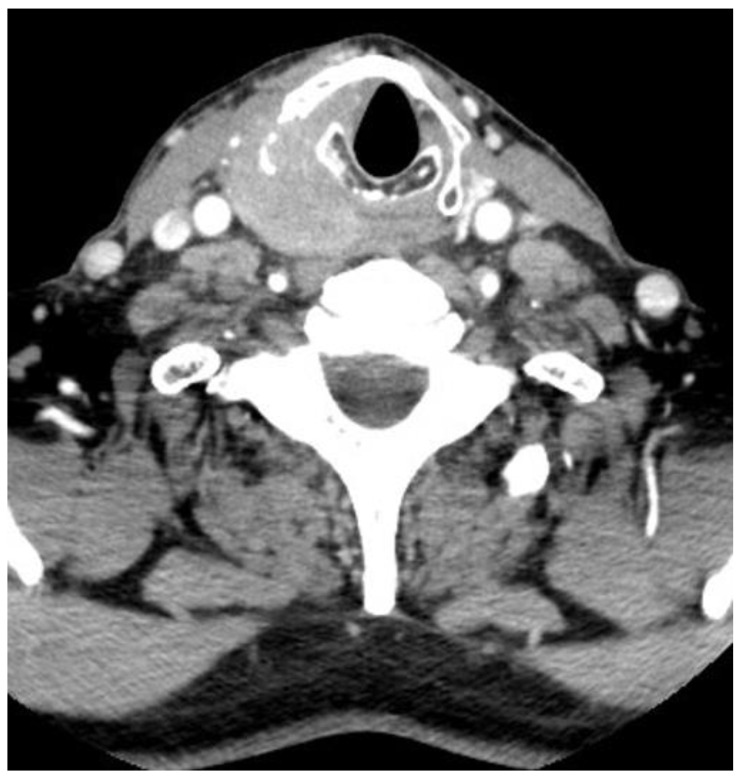
CT scan of a 67-year-old male patient with a T4aN0 hypopharyngeal carcinoma.

Several retrospective studies conducted in patients with laryngeal cancer have demonstrated that T4a primary tumors (extralaryngeal extension with thyroid or cricoid cartilage invasion) were associated with a high risk of treatment failure and local recurrence after nonsurgical treatments [60,61,62]. Therefore, for most authors and therapeutic guidelines, T4a laryngeal cancers are still referred to primary TL [29]. There are few studies evaluating the results of LP therapeutic strategies in patients with T4a HC [29,63]. However, in most centers, patients with T4a HC are considered poor candidates for LP and are managed with primary radical surgery followed by adjuvant RT or CRT (Figure 3). In this regard, in a large, 20-year population-based study conducted in the Netherlands, Petersen et al. showed that, for T3 primary tumors, OS was equal for radical surgery and LP strategies, with a shift in treatment preference between 1991 and 2010 towards organ preservation therapies [63]. However, in this study, OS was significantly better after primary TL (±adjuvant radiotherapy) for T4 primary tumors [63]. In another recent retrospective study in 71 patients with T4 laryngeal or HC treated with either CRT (*n* = 39) or radical surgery (*n* = 32), Al-Mamgani et al. showed that TL resulted in a significantly better local control rate compared to CRT and that the larynx dysfunction-free survival was worse in patients with poor pretreatment laryngeal function [64].

The tumor invades the right piriform sinus, the right part of the thyroid cartilage, and the extralaryngeal spaces.

Prediction of post-therapeutic laryngeal function in patients enrolled in LP programs is difficult and requires great clinical experience. There are no absolute criteria to define an irreversible loss of laryngeal functions. However, pretreatment tracheostomia, swallowing dysfunction with enteral nutrition before therapy, and large cartilage invasion have been shown to be associated with poor functional outcomes in LP studies and are important to consider in the therapeutic decision-making process [15,29]. On the other hand, reversible airway obstruction can be observed in patients with large T3 exophytic tumors. These types of patients can be good candidates for LP even if a pretreatment tracheostomia was required. In these cases of pretherapeutic severe but reversible functional impairment, an ICT-based therapeutic strategy may provide significant benefit compared to definitive CRT, by offering a rapid clinical tumor response. In selected cases, patients can be decannulated after 2 to 3 cycles of TPF-ICT before initiating RT.

Despite the clear shift of the therapeutic paradigm in patients with locally advanced HC in favor of nonsurgical therapeutic strategies aiming at LP, attention should be paid to preserving oncologic outcomes and not compromising survival in patients that are not fit enough to receive a complete therapeutic regimen. Indeed, patients with contraindication for cisplatin-based CRT (poor renal function for example) or TPF-ICT should be referred to primary radical surgery and not to RT alone [15,29]. RT alone is an acceptable therapeutic option only in patients who are unfit to receive CT but refuse TL. When patient tolerance to TPF-ICT is questionable but with no contraindication for cisplatin-based CRT, primary definitive CRT is probably the best therapeutic option compared to a modified ICT regimen. Indeed, ICT-based therapeutic strategies have never demonstrated a clear superiority compared with definitive CRT in HNC patients [57,65].

## 6. Management of Patients with Recurrent and/or Metastatic Disease

Surgery is the standard treatment for patients with isolated local and/or regional tumor recurrence [14,66]. Exceptionally, in highly selected patients, partial open or transoral pharyngolaryngectomy is still feasible. However, in most patients, and particularly in those who relapse after an LP program, radical surgery with TL represents the only available curative therapeutic option [29,66]. However, salvage TL in patients with recurrent HC is associated with a high postoperative morbidity (risk of fistula formation around 50%) and poor survival rates [66,67]. Indeed, compared with other head and neck subsites, recurrent HCs are associated with the lowest rate of successful surgical salvage [66]. In a retrospective analysis of 21 patients who underwent salvage TL with circular pharyngectomy and radial forearm free flap reconstruction, conducted in two French tertiary referral centers, Fakhry et al. showed 2 and 5-year OS rates of 40% and 16%, respectively [67]. This highlights the importance of a careful selection of patients susceptible to benefit from salvage surgery. In a multicenter retrospective cohort study analyzing oncologic outcomes after salvage TL in 405 patients with recurrent laryngeal or HC, Meulemans et al. showed that increasing clinical tumor stage of the recurrent primary tumor, increasing number of metastatic cervical lymph nodes retrieved during neck dissection, hypopharyngeal and supraglottic tumor location, positive section margin status and perineural invasion were independent negative prognostic factors for OS, DSS, and DFS [68].

Patients with metastatic disease or with locoregional recurrence inaccessible to salvage local therapy are treated with systemic therapies according to their general health status (performance status), comorbidities, previous treatment, PD-L1 tumor expression, tumor evolutivity and symptoms [69]. However, local therapy (surgery, stereotactic RT, radiofrequency ablation) has to be considered for patients with a single metastatic or oligometastatic disease [70]. There is no specificity for HC patients compared to patients with other HNC regarding the indications of systemic treatments for metastatic disease. The combination of cisplatin and cetuximab with 5-fluorouracil (EXTREME) or docetaxel (TPEx) are two standard systemic therapy regimens [71]. Alternatively, pembrolizumab (anti-PD1) can be used (alone or with CT: cisplatin + 5-FU) as first-line therapy since a phase III randomized study demonstrated improved OS with pembrolizumab alone in patients whose tumor expresses PD-L1 (combined positive score: CPS ≥ 1) or with pembrolizumab + CT independently of PD-L1 tumor expression [72]. The advent of anti-PD1 has improved survival for metastatic HNC patients, however, it raises the important question of the therapeutic sequence and complicates the decision-making process of medical oncologists in this noncurative setting [69].

## 7. Summary of Oncologic and Functional Outcomes

Patients with early-stage HC have relatively favorable survival outcomes [16,18,19,20,21,23,24,25]. Whatever the therapeutic modality, 5-year OS rates between 60 and 70% are generally reported, which is, however, inferior to the prognosis of early-stage tumors from other head and neck subsites [16,18,19,20,21,23,24,25]. Long-term LP rates are superior to 75%, and most patients recover with a full oral diet [18,19,20,21,23,24,25]. Partial surgery (open, endoscopic, or transoral surgery) exposes the patients to a moderate risk of aspiration or penetration depending on the surgical procedure [16,17,18,19,20,21]. Voice is not affected by the surgical procedure if the integrity and mobility of the vocal cord are not altered. Patients receiving RT alone are exposed to a higher risk of salivary dysfunction (dryness, sticky saliva), which is, together with the reduction of pharyngeal sensitivity and motility, likely to impact swallowing function [23,24,25]. However, xerostomia, dysgeusia, and dental problems are less frequent than for oral or oropharyngeal carcinoma [23,24,25].

Overall, patients with locally advanced HC have a worse prognosis and are exposed to a higher risk of persistent toxicities that can affect their QoL. Whatever the primary treatment modality (primary TL or LP program), 5-year OS rates close to 35% have been reported. In comparison, patients with locally advanced laryngeal cancer display 5-year OS rates generally superior to 50%. For patients included in an LP program, LP rates of approximately 60% have been reported in long-term HC survivors, which is also inferior to those reported in patients with laryngeal carcinoma (about 75% in recent studies).

Dysphagia is the main persistent complaint in locally advanced HC patients [11,12,35]. More than 6 months after therapy, retrospective series have shown a rate of feeding tube dependence of approximately 15%, which is superior to enteral feeding dependence rates reported in patients with locally advanced tumors from other head and neck subsites [11,12,13,35]. However, more favorable swallowing outcomes have been found for the patients who can be included in ICT-based LP programs [12,35]. Multidisciplinary management of swallowing disorders (nutritional counseling, nutritional interventions, speech therapy, etc.) is essential in the long-term follow-up of HC patients since persistent dysphagia has been associated with a higher risk of depression and poor QoL outcomes [73].

Comparison of QoL outcomes according to the primary treatment modality in patients with locally advanced HC is difficult and no significant difference has been reported regarding global QoL scores between patients treated by primary radical surgery and those included in an LP program [9,49]. However, higher social, sensory, and speech problems are usually reported in patients undergoing primary TL whereas higher salivary and teeth problems are generally found in those receiving nonsurgical therapies for LP [9,49]. Psychological distress with persistent anxiodepressive symptoms has been demonstrated in approximately one-third of locally advanced HC survivors, which may affect, along with dysphagia, patient QoL [73,74,75].

## 8. Conclusions

HC remains a life-threatening malignancy and requires complex multimodal management that is best achieved within experienced multidisciplinary teams. Comprehensive patient information is essential, as HC treatment significantly affects important functions as well as patient QoL. Highly selected patients can benefit from conservative surgery with modern surgical approaches such as TORS. However, most patients are diagnosed with a locally advanced tumor that cannot be treated surgically without TL. Patients with a T3 primary tumor and a good general condition are usually candidates for an LP program. Radical surgery with TL, which may involve complex pharyngeal reconstructive procedures, is still indicated as a primary treatment modality, especially for patients with T4a primary tumor, or more commonly, as salvage treatment.

## Data Availability

Not applicable.
